# Dimensionality of the Swahili version of the General Health Questionnaire (GHQ-12) in a Kenyan population: A confirmatory factor analysis

**DOI:** 10.1017/gmh.2024.46

**Published:** 2024-04-11

**Authors:** Dharani Keyan, Dusan Hadzi-Pavlovic, Aemal Akhtar, Katie Dawson, Phiona Naserian Koyiet, Richard Bryant

**Affiliations:** 1 School of Psychology, Science, UNSW Sydney, Sydney, NSW, Australia; 2 School of Psychiatry, Medicine, UNSW Sydney, Sydney, NSW, Australia; 3 Department of Clinical Neuroscience, Division of Insurance Medicine, Karolinska Institutet, Solna, Sweden; 4 Mental Health and Psychosocial Support, World Vision International, Nairobi, Kenya

**Keywords:** confirmatory factor analysis, factor structure, GHQ-12, Kenya, mental health disorder

## Abstract

The current study evaluated the Kiswahili version of General Health Questionnaire (GHQ-12) in a Kenyan context comprising of women exposed to gender-based violence. Participants were randomly drawn from community sampling using household screening methods in peri-urban areas in Nairobi. A total of 1,394 participants with varying levels of literacy (years of education: mean [M] = 9.42; standard deviation [SD] = 3.73) and aged between 18 and 89 years were recruited for the study. The observed factor structure of the GHQ-12 was evaluated using six most tested models querying the dimensionality of the instrument insofar as the impacts of positive and negative wording effects in driving multidimensionality. Results from the confirmatory factor analysis supported a bifactor model, consisting of a general distress factor and two separate factors representing common variance due to the positive and negative wording of items. Overall, the findings support the use of the Kiswahili version of the GHQ-12 as a unidimensional construct with method-specific variance owing to wording effects. Importantly, GHQ-12 responses from a sample of Kenyan women with relatively low levels of literacy are congruent with the factor structure observed in other cross-cultural settings in low- and-middle-income countries.

## Impact statement

The highest prevalence rates of common mental disorders are found in low- and middle-income country settings. The need to have efficacious mental health screening tools to detect psychological problems in large numbers of people is important to address mental health needs. As part of a study looking at the impact of a peer-delivered psychological intervention for Kenyan women exposed to gender-based violence, we used the General Health Questionnaire (GHQ-12) to detect general psychological distress. As the factor structure of the Kiswahili version of the GHQ-12 has yet to be validated in this population, we conducted a series of confirmatory factor analyses to elucidate the underlying factor structure. The findings support the proposition that the GHQ-12 can be interpreted as a unidimensional construct with observed factor variation likely arising from wording effects of the items. Importantly, the current findings support and further extend the previous validation completed in a Kenyan context.

## Introduction

Increased exposure to humanitarian crises worldwide has left many people at a heightened risk of developing common mental disorders (CMDs), including depression, anxiety and post-traumatic stress disorder. Individuals with CMDs suffer from wide-ranging physical health problems in ways that contribute to considerable functional impairment and disability (Whiteford et al., 2013; Patel et al., 2016). The heightened prevalence of CMDs among health problems in low- and middle-income countries (LMICs) (GBD, 2022) is not surprising given that relative allocation of global health resources for mental health toward LMICs pales in comparison to high-income countries (Liese et al., 2019). There are apparent challenges in addressing this burden when varied cultural expressions of CMDs and lack of locally validated screening tools impede rapid identification of individuals in need of care and in turn access to appropriate services. For example, CMDs, including major depression, account for the highest burden of disease in Kenya, a trend commonly evidenced across sub-Saharan Africa (Murray et al., 2012). Yet, when screening tools developed within Western settings are readily used in culturally diverse settings (without prior local validation), the extent of detection bias in observed prevalence rates is unclear. As such, there is a need to use locally validated screening tools to document the prevalence of CMDs more accurately, inform intervention developments, and, in turn, more effectively allocate limited mental health resources in LMICs such as Kenya.

The first step in this endeavor has often involved the translation of screening tools developed in high-income countries. For example, the 12-item version of the General Health Questionnaire (GHQ-12) is a widely used self-administered screening tool aimed at detecting general psychological distress (Goldberg et al., 1997). The brevity of the GHQ-12 has afforded its administration across clinical and community settings, and this has been done through translation into 38 different languages across several cultural contexts including Africa, Asia, Middle East and Latin America (El-Metwally et al., 2018). Yet, the underlying factor structure of the responses generated from this instrument continues to be debated. The GHQ-12 was originally proposed as a unidimensional measure of psychological distress (Goldberg and Williams, 1988) comprising of equal number of positively and negatively worded items that produce a global distress score. Given the inconsistent evidence for a single-factor structure, alternative two-dimensional and three-dimensional models have been proposed as being more suitable. The two-factor model by Andrich and van Schoubroeck (1989) stipulates factors based on wording comprising of “social dysfunction” (all negatively phrased items) and “anxiety/depression” (all positively worded items). The three-factor model proposed by Graetz (1991) is similar to this with the exception that the negatively phrased items have been split into two distinct factors, namely “loss of confidence” and “anxiety/depression.” This three-factor model by Graetz (1991) has evidenced better fit relative to the one-factor unidimensional model across varying studies in languages other than English (Shevlin and Adamson, 2005; Tomás et al., 2017). Yet, one argument against multidimensionality is that distinct factors formed based on wording of items may represent artifacts of individual response bias instead of theoretically distinct factors. To investigate this hypothesis, wording effects of the GHQ-12 have been modelled in varying ways. First, Hankins (2008) explored a unidimensional model that accounted for wording effects (with two- and three-dimensional models) by correlating error terms of the negatively phrased items to capture systematic error variance, termed a “response bias” model. Second, Ye (2009) extended this approach and modelled an orthogonal (i.e., uncorrelated) method factor associated with negative items, in addition to a general distress factor, termed “method factor” model. Finally, Tomás et al. (2017) extended this line of thinking, where two orthogonal method factors are modelled to incorporate negatively and positively phrased items, in addition to the single general distress factor, and termed this as a “bifactor” model. The advantage of this bifactor model has allowed researchers to evaluate common variance among a set of items that can be accounted for by a single unidimensional factor, in addition to variance accounted for by method-specific factors (Reise et al., 2007), not allowed by models proposed by Hankins (2008) and Ye (2009). For example, Centofanti et al. (2019) demonstrated the presence of a strong general distress factor as evidenced by the total amount of observed score variance (i.e., omega hierarchical value of 0.81). Yet, the literature is inconsistent as some studies have shown that the multidimensional model by Graetz (1991) outperforms the bifactor model even after taking wording effects into account (Abubakar and Fischer, 2012; Tomás et al., 2017). The picture is further complicated when findings across studies cannot be easily compared within the context of different scoring methods of the GHQ-12 (Tomás et al., 2017) potentially influencing the observed factor structure and model fit (Rey et al., 2014; Centofanti et al., 2019).

Further, very little is known about the specific factor structure of the Kiswahili (or Swahili) version of this instrument. Investigating the factor structure within this specific context has both practical and theoretical importance. Firstly, Kiswahili is an East African language spoken in more than 14 countries with over 200 million speakers (Lisanza, 2021). As such having the Kiswahili version of the GHQ-12 validated will have wide-ranging practical implications for the selection of locally appropriate screening tools insofar as identification of CMDs in low-resourced sub-Saharan African regions. Second, translation of Western instruments to local languages often brings with it the challenge of finding appropriate wording considered to be equivalent to the Western constructs used in the original instrument. Variations in wording of self-report instruments can often pose unintended consequences in terms of interfering with the subsequent latent structure analysis (Tomás et al., 2013). This is particularly relevant for the context surrounding the use of the Kiswahili version, where psychological distress is commonly expressed through use of local idioms and somatic symptoms (Patel, 1998; Patel and Kleinman, 2003), in turn posing potential variability for the dimensionality of the instrument. The current state of the evidence suggests that the wording of items in the GHQ-12 can indicate differences in response bias or theoretically distinct constructs depending upon the demographic of the sample studied and type of scoring method used. Together, these concerns in the literature motivated the current study to examine the factor structure of Kiswahili version of the GHQ-12 and identify the best fitting model.

The purpose of the present study was to analyze the factor structure of the Kiswahili version of the GHQ-12 administered in a Kenyan context. To the best of our knowledge, one study has previously investigated the factor structure of the GHQ-12 in Kenya (Abubakar and Fischer, 2012); however, this study administered the instrument in a literate English-speaking population of adolescents and adults and did not explore specific reliability estimates (i.e., omega values). We aimed to contribute to the existing debate relating to the dimensionality of the GHQ-12 by choosing models that have garnered the most investigation insofar as ascertaining the extent of wording effects on subsequent observed dimensionality (Abubakar and Fischer, 2012; Hystad and Johnsen, 2020). The current study differs from these previous studies in several ways. We administered the Kiswahili version of the GHQ-12 to a sample of Kenyan women exposed to gender-based violence. Participants were drawn from the community via random household screening in peri-urban Nairobi and thus comprised of varying levels of literacy. This makes our study the first to explore factor structures and associated method effects within an LMIC setting. Here, we evaluate the reliability of the best-fitting model through calculation of omega estimates to better understand the observed factor structure and verify accompanying fit indices, and this, to date, has not been assessed in the target population. To this end, the present study aimed at exploring two research questions: (a) does the Kiswahili version of the GHQ-12 display the same factor structure in a Kenyan population as evidenced in previous studies (Abubakar and Fischer, 2012) and (b) to what extent is the factorial structure of GHQ-12 (in a sample of Kenyan women with varying levels of literacy) influenced by wording effects.

## Methods

### Participants and procedure

The study was carried out in the Dagoretti Sub County, Nairobi, in Kenya. Data were collected across three local health care facilities that are part of the primary health care system. This sample was screened for participation in a larger study on women affected by adversity and gender-based violence in urban areas in Nairobi (Sijbrandij et al., 2016; Bryant et al., 2017). Participants were recruited through random community screening at their households by independent assessors. Households were selected using a population-based interval approach, where members of a larger population were selected according to a random starting point, and a fixed periodic interval. This is called the sampling interval and it is determined by dividing the population size by the desired sample size. Further details on sampling methods have been detailed elsewhere (Sijbrandij et al., 2016). Following selection of households, independent assessors approached the head of each household and asked to interview a random adult woman aged 18 or older. Informed consent was obtained by giving each person 24 h to consider their decision to partake in the trial. For those women who were illiterate, oral consent and a thumb print in lieu of a signature were obtained with an independent witness, in line with WHO recommendations (Bhutta, 2004). Baseline screening for general distress was then administered by an independent assessor at participants’ households or a private space near their homes. If a household declined to be screened and/or did not have a woman aged 18 years or older, the independent assessor moved to the next household based on the predetermined sampling interval. Inclusion criteria for this study required that participants were (a) females and (b) over 18 years of age. Data for the GHQ-12 in the current study are based on screening surveys conducted in a total of 1,394 participants.

### Measures

#### GHQ-12

The GHQ-12 is a well-validated indicator of general psychological distress (Goldberg and Williams, 1988) that was used for screening eligible participants in the current study. The GHQ-12 comprises 12 questions about general well-being, experience of depressive and anxiety symptoms and sleep disturbances over “the past few weeks.” In the current study, items were scored on a 4-point Likert scale ranging from 0 to 3 (range 0 to 36; higher scores indicate severe psychological distress). The GHQ-12 was translated into Kiswahili for the current study and was used to detect psychological comorbidity, as has been previously used in Kenya (Getanda, Papadopoulos, and Evans, 2015).

Prior to screening, this GHQ-12 was translated through a process of cultural adaptation involving two workshops with mental health experts, translators and community health workers. Adaptation of measures focused on cultural appropriateness in terms of language, metaphors, content, concepts and context in line with established guidelines (Bernal and Sáez-Santiago, 2006).

### Data analysis plan

**Figure 1.**
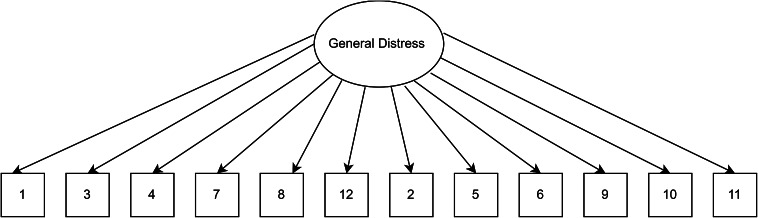
Unidimensional model.

**Figure 2.**
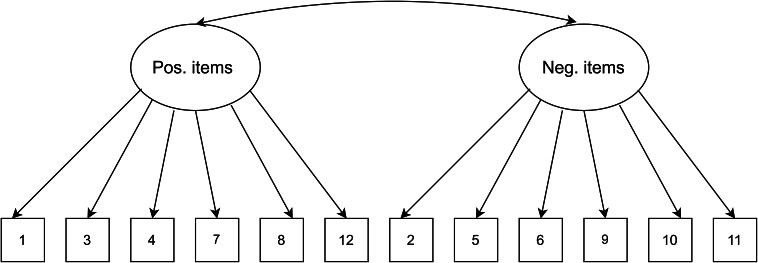
Two-dimensional model.

**Figure 3.**
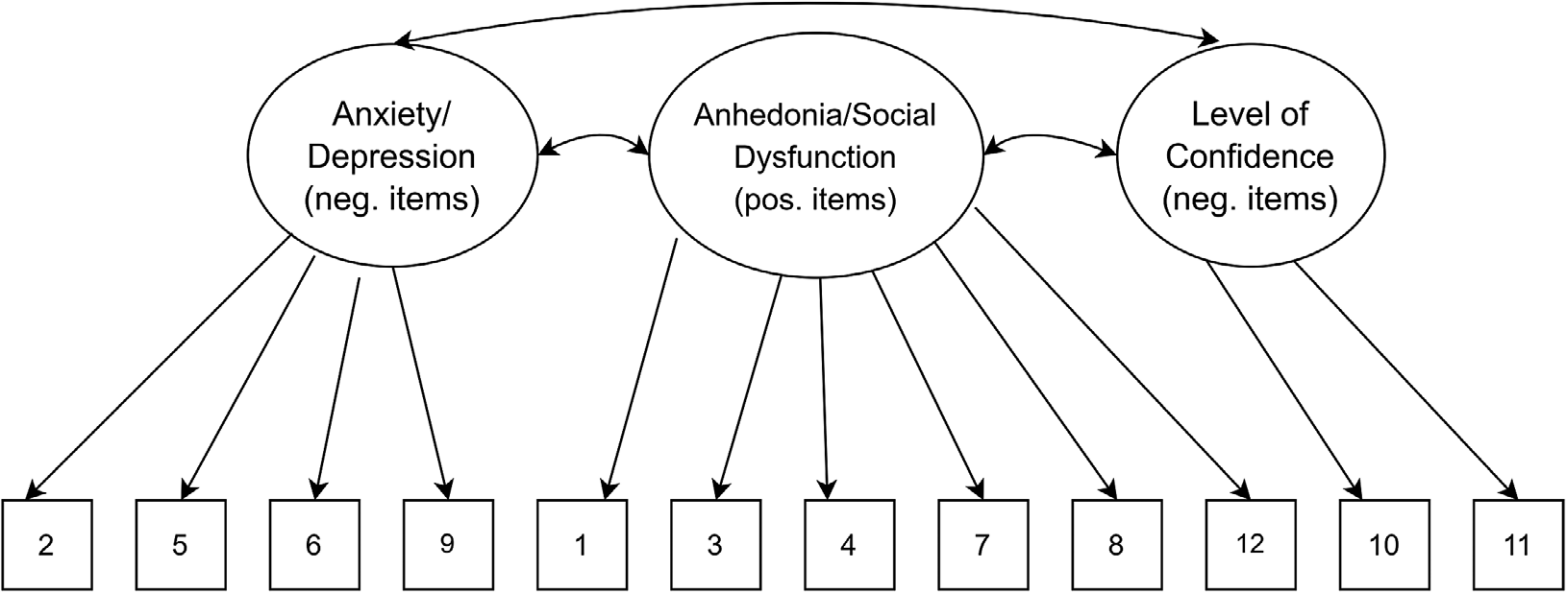
Three-dimensional model.

**Figure 4.**
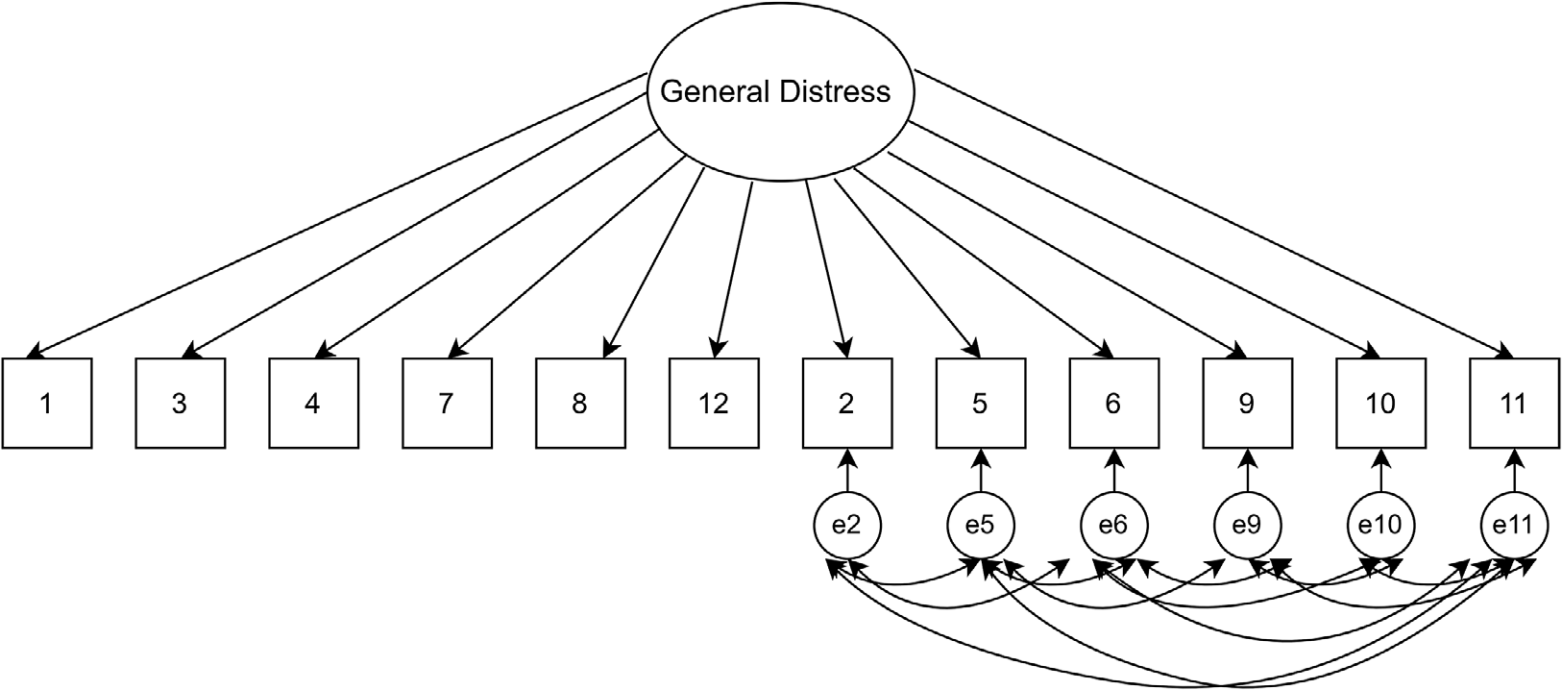
Response bias model.

**Figure 5.**
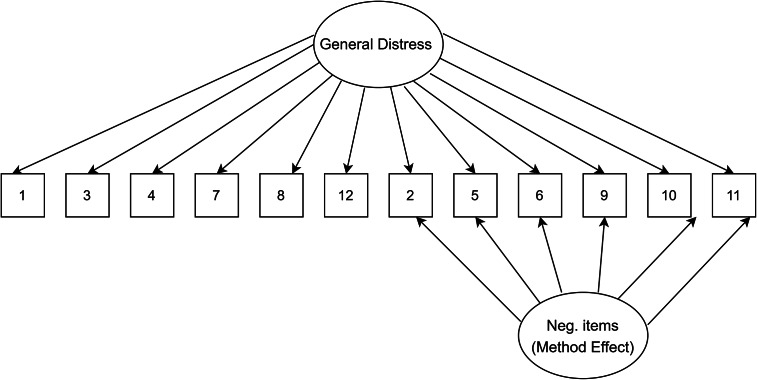
Method factor model.

**Figure 6.**
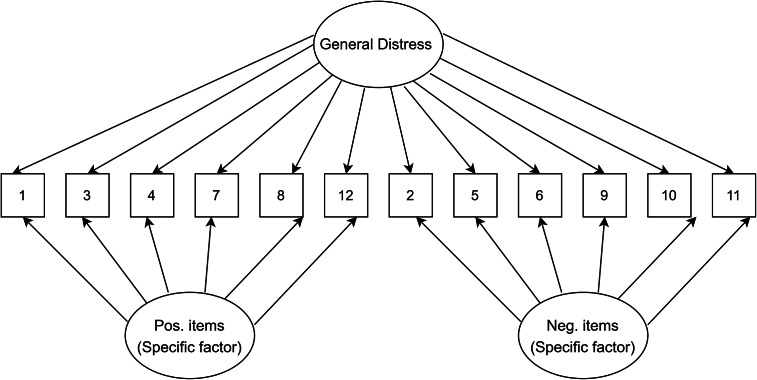
Bifactor model.

A series of planned confirmatory factor models were examined using MPLUS8 (Muthén and Muthén, 1998–2018) and R packages, including lavaan and psych (Rosseel, 2012). A priori models were estimated using the maximum likelihood estimation (Bentler and Chou, 1987), where this method has been previously used for the 4-point Likert scoring system of the GHQ-12 (King et al., 2023). Other previous studies have also used the variance-adjusted weighted least squares (WLSMV) (Flora and Curran, 2004; Wirth and Edwards, 2007) for ordered categorical items; we carried out analyses with this estimation method as a check for sensitivity, and these WLSMV estimates are available in the Supplementary material. First, our baseline model consisting of a unidimensional model with a single factor as originally stipulated was assessed (see Figure 1). To explore whether the observed responses from the Kiswahili version of the GHQ-12 represented distinct theoretical factors, models 2 and 3 were chosen (see Figures 2 and 3). Specifically, a two-factor model as proposed by Andrich and van Schoubroeck (1989) was assessed where one factor included all negatively-worded items and the second factor encompassing all positively worded items. Additionally, a three-factor model proposed by Graetz (1991) was tested. This model was similar to the two-factor model, with the exception that the negatively phrased items were split into two distinct factors: (1) loss of confidence and (2) anxiety and depression. To explore the role of wording effects and related response biases, we chose models 4 to 6. Specifically, model 4 investigated a unidimensional model where errors of all the negatively worded items were correlated as proposed by Hankins (2008) (see Figure 4). Moreover, model 5 explored a unidimensional model with an orthogonal (i.e., uncorrelated) method factor for the negatively worded items, as proposed by Ye (2009) (see Figure 5). Finally, model 6 is an extension of model 5, whereby an orthogonal factor for positively phrased items was included (see Figure 6). Also referred to as a bifactor model, this model has been previously tested (Tomás et al., 2017). This bifactor model was chosen due to its validation with the GHQ-12 in recent times (Centofanti et al., 2019) and thus provides an extension of previous validation studies in a Kenyan context (Abubakar and Fischer, 2012).

Individual model fit will be evaluated by examining a combination of the size and statistical significance of factor loadings as well as several commonly used goodness-of-fit statistical indices suggested in the extant literature. Specifically, various parameters including Comparative Fit Index (CFI), root mean square error of approximation (RMSEA) and Tucker–Lewis index (TLI) were chosen. Good model fit has been shown to be reflected in CFI and TLI values greater than or equal to 0.95 (Hu and Bentler, 1999). Additionally, RMSEA values reflect sensitivity to model mis-specification where values closer to 0.06 have been proposed to indicate good model fit. As the chi-square metric is known to be sensitive to sample size, it was used in combination with the above fit indices (Schumacker and Lomax, 2004). Additionally, comparative model fit will be examined with two measures including the Akaike information criterion and the Bayesian information criterion, where lower values for both measures indicate better fit. Finally, to explore the reliability of observed dimensionality, we computed omega estimates including omega hierarchical (ω_h_, proportion of total score variance owing to general factor over and above specific factors) and omega total (ω, proportion of total variance across general and specific factors) scores (McNeish, 2018).

## Results

### Demographic characteristics

Data were collected from 1,394 adult females aged 18 years and above, with a mean age of 32.82 years (standard deviation [SD] = 11.539, range = 18–89). Years of education ranged from 0 to 24 years (mean [M] =9.42, SD = 3.73). Specifically, 11% of women had five or less years of education and 60% had 10 or less years of education.

### Confirmatory factor analyses

Table 1 presents fit indices of the models assessed. The single-factor unidimensional model (Figure 1) was initially tested, and fit indices indicated that this model provided the poorest fit of all the models tests (CFI = 0.902; TLI = 0.878; standardized root mean square residual [SRMR] = 0.050; RMSEA = 0.093 with 90% confidence interval [CI] for RMSEA = 0.087 - 0.099). The two- (Figure 2) and three-dimensional (Figure 3) models (without method effects) improved these fit statistics, where the latter provided more acceptable fit on the RMSEA (RMSEA = 0.046 with 90% CI for RMSEA = 0.040–0.053). After adjusting for correlated errors between items, the response bias model (Figure 4) had a similar fit to the three-dimensional model (RMSEA = 0.047 with 90% CI for RMSEA = 0.040–0.055). The method factor model (Figure 5) with the artifactual factor containing all the negative items did not fit the data better than the three-dimensional model (CFI = 0.953; TLI =0.935; SRMR = 0.034; RMSEA = 0.068 with 90% CI for RMSEA = 0.061–0.075). The bifactor model (Figure 6) provided good fit statistics on all indices (See Table 1). The standardized factor loadings for the bifactor model are presented in Table 3, and for all other models in Table 2. All absolute residual correlations were small for the bifactor model (<0.04).

**Table 1.** Model fit indices

Model	*χ*2	df	*p* value	AIC	BIC	CFI	TLI	RMSEA (90% CI)	SRMR
Unidimensional	682.179	53	0.000	35,419.19	35,612.43	0.902	0.878	0.093 (0.087–0.099)	0.050
Two-dimensional	408.204	53	0.000	34,942.10	35,145.78	0.945	0.931	0.070 (0.064–0.076)	0.037
Three-dimensional	201.089	51	0.000	34,942.10	35,145.78	0.977	0.970	0.046 (0.040–0.053)	0.028
Response bias (correlated errors)	158.317	39	0.000	34,923.33	35,189.68	0.981	0.969	0.047 (0.040–0.055)	0.026
Method factor	350.211	48	0.000	35,097.22	35,316.57	0.953	0.935	0.068 (0.061–0.075)	0.034
Bifactor	132.940	42	0.000	34,891.95	35,142.64	0.986	0.978	0.040 (0.032–0.047)	0.020

AIC, Akaike information criterion; BIC, Bayesian information criterion; CI, confidence interval; CFI, Comparative Fit Index; RMSEA, root mean square error of approximation; SRMR, standardized root mean square residual; TLI, Tucker–Lewis index.

**Table 2.** Standardized factor loadings for the alternative models of the GHQ-12

		Model 1	Model 2		Model 3			Model 4	Model 5		Model 6		
Items			I	II	I	II	III	I	I	II	I	II	III
1	Able to concentrate	0.572	0.628			0.626		0.624	−0.620		0.518	0.340	
2	Loss of sleep over worry	0.698		0.718	0.724			0.558	−0.563	0.461	0.704		0.136
3	Playing a useful part	0.480	0.537			0.536		0.535	−0.530		0.419	0.394	
4	Capable of making decisions	0.378	0.457			0.458		0.458	−0.454		0.308	0.453	
5	Felt constantly under strain	0.757		0.781	0.802			0.612	−0.606	0.536	0.771		0.290
6	Could not overcome difficulties	0.754		0.765	0.774			0.649	−0.643	0.413	0.750		0.193
7	Able to enjoy day–to–day activities	0.606	0.683			0.684		0.686	−0.682		0.549	0.407	
8	Able to face problems	0.627	0.666			0.664		0.664	−0.658		0.581	0.273	
9	Feeling unhappy and distressed	0.768		0.789	0.799			0.629	−0.632	0.487	0.776		0.162
10	Losing confidence	0.637		0.633			0.755	0.577	−0.609	0.182	0.695		−0.397
11	Thinking of self as worthless	0.672		0.674			0.800	0.582	−0.618	0.246	0.715		−0.267
12	Feeling reasonably happy	0.598	0.653			0.656		0.656	−0.658		0.554	0.321

**Table 3.** Standardized factor loadings and variance composition for the bifactor model of the General Health Questionnaire (GHQ-12)

	Model 6: Bifactor Model		
	I	II	II
Able to concentrate	0.518	0.340	
Loss of sleep over worry	0.704		0.136
Playing a useful part	0.419	0.394	
Capable of making decisions	0.308	0.453	
Felt constantly under strain	0.771		0.290
Could not overcome difficulties	0.750		0.193
Able to enjoy day–to–day activities	0.549	0.407	
Able to face problems	0.581	0.273	
Feeling unhappy and distressed	0.776		0.162
Losing confidence	0.695		−0.397
Thinking of self as worthless	0.715		−0.267
Feeling reasonably happy	0.554	0.321	
ECV	0.850	0.073	0.078
Ω	0.906		
ω_s_		0.783	0.892
ω_h_	0.832		
ω_hs_		0.281	0.001

ECV, explained common variance; ω, omega; ω_s_, omega subscale; ω_h_, omega hierarchical; ω_hs_, omega hierarchical subscale.

To explore the amount of observed score variance accounted for by the general factor relative to specific factors of the bifactor model, different forms of omega estimates were computed. First, the omega (ω) value is a reliability estimate of a unit-weighted total score of all GHQ items representing both the general and specific factors and is based on the sums of squared loadings and error variances. Here, approximately 91% of variance in this total scale score is accounted for by the combination of the general and specific factors (ω = 0.906). Specifically, approximately 83% of the variance in the observed scale score is attributed to just the general factor (omega hierarchical; ω_h_ = 0.832). After controlling for the general factor, approximately 28% of the variance in the positive subscale score is accounted for by the specific factor (Omega hierarchical subscale; ω_hs_ = 0.281), and approximately 0.1% variance accounted for by the negative subscale score for the remaining specific factor (ω_hs_ = 0.001). As seen in Table 3, it is worth noting that omega hierarchical values (ω_hs_) were very low relative to the omega subscale scores (ω_s_), suggesting that a large proportion of the variance in subscale scores can be attributed to the general factor as opposed to unique specific factor variances.

## Discussion

This study examined the factorial structure of the Kiswahili version of the GHQ-12 in a sample of Kenyan women exposed to gender-based violence. To our knowledge, this is the first study to assess the five alternative models proposed in the literature. Our findings indicate that the GHQ-12 factor structure as derived from a sample of Kenyan women with varying levels of literacy displays a general distress factor with two separate factors resulting from method-specific wording effects. This is similar to recent validation of the GHQ-12 within other demographic samples comprising of literate English-speaking individuals (Centofanti et al., 2019; Hystad and Johnsen, 2020). Importantly, the current findings support the proposition that variation in factors across multidimensional models (i.e., two- and three-factor models) may be largely related to differential phrasing of items. Specifically, the finding that negatively and positively worded items load onto separate factors is consistent with suggestions of method-specific variance in the GHQ-12 that likely drives its observed multidimensionality (Hankins, 2008; Ye, 2009. The current study extends previous support for the unidimensional GHQ-12 in an English-speaking literate Kenyan population (Abubakar and Fischer, 2012) by considering both positive and negative wording effects (Marsh et al., 2010). In the current analysis, the general factor accounted for nearly 91% of the total score variance (omega[ω] = 0.906), while the specific method factors accounted for very little in comparison, after controlling for the general factor variance (omegas = 0.001–0.281). It is worth noting that the three-dimensional model displayed a good fit that was comparable to the bifactor model, suggesting that the plausibility of the three-dimensional model cannot be precluded based on observed fit indices alone. However, the practical utility in having three factors is questioned within the context of high correlations among the three factors and high standardized factor loadings for the latent factors. Here, our sensitivity analyses using the WLSMV estimator evidence similar conclusions regarding the bifactor and three-factor models (see Supplementary material). To this end, previous research has questioned the discriminative power among the three factors and suggested that the three factors do not afford additional predictive power (e.g., Ye, 2009). Further support for this is seen in the goodness-of-fit indices of the two- and three-dimensional models that fell within close range of the unidimensional models fitted with method effects (i.e., models 4 and 5), in turn suggesting little incremental value for having theoretical distinct factors. Importantly, we followed up the observation that the bifactor model may offer the most sufficient explanation of the GHQ-12 through computation of specific omega estimates. Taken together, we find that the bifactor model comprising of a single general distress factor and two separate factors capturing method-specific variance offers the most parsimonious explanation of the GHQ-12 factor structure. Here, it is worth considering that summation of items on the GHQ-12, as commonly scored, may reflect the influence of item phrasing that is beyond the dominant general distress factor.

Nonetheless, the current findings offer important practical relevance for administering the GHQ-12 in cross-cultural settings. From an applied perspective, the findings suggest that the Kiswahili version of the GHQ-12 may be administered in a Kenyan population and interpreted in a similar manner to the English version of this measure administered in a demographical similar population (Abubakar and Fischer, 2012). Here, screening for general distress including symptoms of CMDs including anxiety and depression as measured by the GHQ-12 in this setting offers similar clustering as compared to other cultural settings around the world (Tomás et al., 2017). This is an important reflection as some research has previously suggested that clustering of items in the GHQ-12 item may differ across cultural contexts (Romppel et al., 2017). However, this proposed functional equivalence of the GHQ-12 requires further statistical verification insofar as performing tests of measurement invariance. Specifically, comparison of the GHQ-12 across literacy groups and across time will provide important information on the predictive utility of the observed response patterns.

To this end, we acknowledge several limitations to the current study. First, the generalizability of our findings needs to be assessed further to mix-gendered samples in the same population. Second, the current analysis was limited to the 4-point Likert rating method of the GHQ-12; here, scoring system can influence the number of factors obtained as well as item-factor loadings (Aguado et al., 2012) with other studies using different scoring systems, including 6-point (Kalliath, O’Driscoll, and Brough, 2004) and 7-point (Ye, 2009) scales. Third, we recognize that this sample was challenged in levels of literacy as measured by years of education, and this may have influenced response patterns insofar as how items are perceived and responded to by the target sample. Fourth, tests of measurement invariance will help further consolidate our proposition that the GHQ-12 can be practically interpreted in ways similar to the English version of this instrument.

These limitations notwithstanding the current findings strengthen support for the proposition that the GHQ-12 is a unidimensional measure with factorial variation that likely arises from wording effects. Our findings are strengthened by reports of omega reliability estimates and this is an extension of the research previously conducted in Kenya (Abubakar and Fischer, 2012). From a practical or applied perspective, it may be acceptable to utilize this instrument as a unidimensional measure within the cultural context of Kenya.

## Supporting information

Keyan et al. supplementary material Keyan et al. supplementary material

## Data Availability

Data and materials will be available on request to the corresponding author, D.K.

## Supplementary material

The supplementary material for this article can be found at http://doi.org/10.1017/gmh.2024.46.
