# Supplementary material for: Dimensionality of the Swahili version of the General Health Questionnaire (GHQ-12) in a Kenyan population: A confirmatory factor analysis
Source: Glob Ment Health (Camb). 2024 Apr 11;11:e48. doi: 10.1017/gmh.2024.46 (PMC11058525; doi:10.1017/gmh.2024.46)
Supplement: Keyan et al. supplementary material [file S2054425124000463sup001.docx]

**Table 1:** Model fit indices

| **Model** | **χ2** | **df** | | **p-value** | **CFI** | **TLI** | **RMSEA (90% CI)** | **SRMR** |
| --- | --- | --- | --- | --- | --- | --- | --- | --- |
| 1. Unidimensional | 1120.12 | 54 | 0.000 | | 0.939 | 0.925 | 0.120 (0.114 - 0.126) | 0.055 |
| 1. Two-dimensional | 501.375 | 53 | 0.000 | | 0.974 | 0.968 | 0.077 (0.071 - 0.084) | 0.036 |
| 1. Three-dimensional | 359.998 | 51 | 0.000 | | 0.982 | 0.977 | 0.065 (0.059 - 0.072) | 0.030 |
| 1. Response bias (correlated errors) | 317.118 | 39 | 0.000 | | 0.984 | 0.973 | 0.070 (0.063 - 0.078) | 0.028 |
| 1. Method factor | 490.99 | 48 | 0.000 | | 0.975 | 0.965 | 0.082 (0.076 - 0.089) | 0.035 |
| 1. Bifactor | 193.68 | 42 | 0.000 | | 0.991 | 0.986 | 0.051 (0.044 - 0.059) | 0.021 |

*AIC,* Akaike information criterion; *BIC,* Bayesian information criterion; *CFI*, comparative fit index; *CI*, confidence interval; *RMSEA*, root mean square error of approximation; *SRMR*, standardized root mean square residual; *TLI,* Tucker-Lewis index.

**Table 2:** Standardized factor loadings for the alternative models of the GHQ-12

|  |  | **Model 1** | **Model 2** | | **Model 3** | | | **Model 4** | **Model 5** | | **Model 6** | | |
| --- | --- | --- | --- | --- | --- | --- | --- | --- | --- | --- | --- | --- | --- |
| Items |  |  | **I** | **II** | **I** | **II** | **III** | **I** | **I** | **II** | **I** | **II** | **II** |
| 1 | Able to concentrate | 0.621 | 0.683 |  |  | 0.683 |  | 0.683 | 0.683 |  | 0.551 | 0.383 |  |
| 2 | Loss of sleep over worry | 0.751 |  | 0.770 | 0.780 |  |  | 0.598 | 0.596 | 0.500 | 0.765 |  | 0.119 |
| 3 | Playing a useful part | 0.525 | 0.573 |  |  | 0.573 |  | 0.573 | 0.573 |  | 0.421 | 0.477 |  |
| 4 | Capable of making decisions | 0.456 | 0.500 |  |  | 0.500 |  | 0.500 | 0.500 |  | 0.326 | 0.539 |  |
| 5 | Felt constantly under strain | 0.825 |  | 0.844 | 0.853 |  |  | 0.650 | 0.650 | 0.558 | 0.835 |  | 0.269 |
| 6 | Couldn’t overcome difficulties | 0.809 |  | 0.830 | 0.841 |  |  | 0.685 | 0.682 | 0.452 | 0.821 |  | 0.174 |
| 7 | Able to enjoy day-to-day activities | 0.684 | 0.745 |  |  | 0.745 |  | 0.745 | 0.745 |  | 0.593 | 0.465 |  |
| 8 | Able to face problems | 0.662 | 0.726 |  |  | 0.726 |  | 0.726 | 0.726 |  | 0.613 | 0.299 |  |
| 9 | Feeling unhappy and distressed | 0.820 |  | 0.842 | 0.853 |  |  | 0.664 | 0.662 | 0.525 | 0.837 |  | 0.110 |
| 10 | Losing confidence | 0.767 |  | 0.793 |  |  | 0.858 | 0.642 | 0.645 | 0.447 | 0.806 |  | -0.446 |
| 11 | Thinking of self as worthless | 0.777 |  | 0.802 |  |  | 0.879 | 0.634 | 0.641 | 0.481 | 0.801 |  | -0.239 |
| 12 | Feeling reasonably happy | 0.650 | 0.709 |  |  | 0.709 |  | 0.709 | 0.709 |  | 0.583 | 0.358 |  |

Model 1= Unidimensional (I = General Distress); Model 2= Two dimensional (I = Positive; II = negative); Model 3 = Three dimensional model (Anxiety/Depression; II = Anhedonia; III = Confidence); Model 4 = Response bias Model (Correlated error terms for negative items); Model 5 = Method factor model (I = General Distress; II = Method factor) ; Model 6 = Bifactor model (I = General Distress; II = Positive method factor; III = Negative Method Factor)

**Table 3:** Standardized factor loadings and variance composition for the Bifactor model of the General Health Questionnaire (GHQ-12)

|  | **Model 6: Bifactor Model** | | |
| --- | --- | --- | --- |
|  | **I** | **II** | **II** |
| Able to concentrate | 0.551 | 0.383 |  |
| Loss of sleep over worry | 0.765 |  | 0.119 |
| Playing a useful part | 0.421 | 0.477 |  |
| Capable of making decisions | 0.326 | 0.539 |  |
| Felt constantly under strain | 0.835 |  | 0.269 |
| Couldn’t overcome difficulties | 0.821 |  | 0.174 |
| Able to enjoy day-to-day activities | 0.593 | 0.465 |  |
| Able to face problems | 0.613 | 0.299 |  |
| Feeling unhappy and distressed | 0.837 |  | 0.110 |
| Losing confidence | 0.806 |  | -0.446 |
| Thinking of self as worthless | 0.801 |  | -0.239 |
| Feeling reasonably happy | 0.583 | 0.358 |  |
| ECV | 0.791 | 0.155 | 0.054 |
| ω | 0.911 |  |  |
| ω_s_ |  | 0.764 | 0.896 |
| ω_h_ | 0.861 |  |  |
| ω_hs_ |  | 0.293 | 0.002 |

ECV= explained common variance; ω = omega; ω_s =_ omega subscale; ω_h =_ omega hierarchical;

ω_hs_ = omega hierarchical subscale
