# Supplementary material for: Dimensionality of the Swahili version of the General Health Questionnaire (GHQ-12) in a Kenyan population: A confirmatory factor analysis
Source: Glob Ment Health (Camb). 2024 Apr 11;11:e48. doi: 10.1017/gmh.2024.46 (PMC11058525; doi:10.1017/gmh.2024.46)
Supplement: Peer review history

# Editor report: Recommendation: Dimensionality of the Swahili version of the General Health Questionnaire (GHQ-12) in a Kenyan population: A confirmatory factor analysis — R0/PR2

**DOI:** 10.1017/gmh.2024.46.pr2

**Affiliations:** Clinical, Neuro- and Developmental Psychology, Vrije Universiteit Amsterdam, Netherlands

Many thanks for submitting this paper to GMH. As you will see, your paper was reviewed by two reviewers, who both noticed significant strengths and also expressed some concerns.

They also mentioned some overlapping points, including a more thorough discussion of the previous literature validating the GHQ-12 in the introduction. I recommend to address all individual concerns raised by each reviewer in case you decide to revise and resubmit your paper to GMH.

Also, the paper would benefit from adding some discussion of the use of these instruments in measuring concepts such as general distress among culturally diverse populations, such as your sample of Kenyan females.

# Editor report: Decision: Dimensionality of the Swahili version of the General Health Questionnaire (GHQ-12) in a Kenyan population: A confirmatory factor analysis — R0/PR3

**DOI:** 10.1017/gmh.2024.46.pr3

**Affiliations:** London School of Hygiene & Tropical Medicine, United Kingdom of Great Britain and Northern Ireland

No accompanying comment.

# Editor report: Recommendation: Dimensionality of the Swahili version of the General Health Questionnaire (GHQ-12) in a Kenyan population: A confirmatory factor analysis — R1/PR5

**DOI:** 10.1017/gmh.2024.46.pr5

**Affiliations:** Clinical, Neuro- and Developmental Psychology, Vrije Universiteit Amsterdam, Netherlands

No accompanying comment.

# Editor report: Decision: Dimensionality of the Swahili version of the General Health Questionnaire (GHQ-12) in a Kenyan population: A confirmatory factor analysis — R1/PR6

**DOI:** 10.1017/gmh.2024.46.pr6

**Affiliations:** London School of Hygiene & Tropical Medicine, United Kingdom of Great Britain and Northern Ireland

No accompanying comment.

# Editor report: Recommendation: Dimensionality of the Swahili version of the General Health Questionnaire (GHQ-12) in a Kenyan population: A confirmatory factor analysis — R2/PR8

**DOI:** 10.1017/gmh.2024.46.pr8

**Affiliations:** London School of Hygiene & Tropical Medicine, United Kingdom of Great Britain and Northern Ireland

No accompanying comment.

# Editor report: Decision: Dimensionality of the Swahili version of the General Health Questionnaire (GHQ-12) in a Kenyan population: A confirmatory factor analysis — R2/PR9

**DOI:** 10.1017/gmh.2024.46.pr9

**Affiliations:** London School of Hygiene & Tropical Medicine, United Kingdom of Great Britain and Northern Ireland

No accompanying comment.
